# Stepwise Evolution of a Klebsiella pneumoniae Clone within a Host Leading to Increased Multidrug Resistance

**DOI:** 10.1128/mSphere.00734-21

**Published:** 2021-11-24

**Authors:** Mai Yoshino, Masamune Aihara, Yasuhiro Gotoh, Masaru Akimoto, Wakana Tatsuhara, Makiko Kiyosuke, Yuichi Matsushima, Takeshi Uchiumi, Tetsuya Hayashi, Dongchon Kang

**Affiliations:** a Department of Clinical Chemistry and Laboratory Medicine, Kyushu University Hospital, Fukuoka, Japan; b Department of Bacteriology, Graduate School of Medical Sciences, Kyushu University grid.411248.agrid.177174.3 , Fukuoka, Japan; c Department of Health Science, Graduate School of Medical Sciences, Kyushu University grid.411248.agrid.177174.3 , Fukuoka, Japan; d Department of Clinical Chemistry and Laboratory Medicine, Graduate School of Medical Sciences, Kyushu University grid.411248.agrid.177174.3 , Fukuoka, Japan; Escola Paulista de Medicina/Universidade Federal de São Paulo

**Keywords:** *Klebsiella*, carbapenems, drug resistance evolution, multidrug resistance

## Abstract

Five *bla*_CTX-M-14_-positive Klebsiella pneumoniae isolates (KpWEA1, KpWEA2, KpWEA3, KpWEA4-1, and KpWEA4-2) were consecutively obtained from a patient with relapsed acute myeloid leukemia who was continuously administered antimicrobials. Compared with KpWEA1 and KpWEA2, KpWEA3 showed decreased susceptibility to antimicrobials, and KpWEA4-1 and KpWEA4-2 (isolated from a single specimen) showed further-elevated multidrug-resistance (MDR) phenotypes. This study aims to clarify the clonality of the five isolates and their evolutionary processes leading to MDR by comparison of these complete genomes. The genome comparison revealed KpWEA1 was the antecedent of the other four isolates, and KpWEA4-1 and KpWEA4-2 independently emerged from KpWEA3. Increasing levels of MDR were acquired by gradual accumulation of genetic alterations related to outer membrane protein expression: the loss of OmpK35 and upregulation of AcrAB-TolC occurred in KpWEA3 due to *ramA* overexpression caused by a mutation in *ramR*; then OmpK36 was lost in KpWEA4-1 and KpWEA4-2 by different mechanisms. KpWEA4-2 further acquired colistin resistance by the deletion of *mgrB*. In addition, we found that *exuR* and *kdgR*, which encode repressors of hexuronate metabolism-related genes, were disrupted in different ways in KpWEA4-1 and KpWEA4-2. The two isolates also possessed different amino acid substitutions in AtpG, which occurred at very close positions. These genetic alterations related to metabolisms may compensate for the deleterious effects of major porin loss. Thus, our present study reveals the evolutionary process of a K. pneumoniae clone leading to MDR and also suggests specific survival strategies in the bacteria that acquired MDR by the genome evolution.

**IMPORTANCE** Within-host evolution is a survival strategy that can occur in many pathogens and is often associated with the emergence of novel antimicrobial-resistant (AMR) bacteria. To analyze this process, suitable sets of clinical isolates are required. Here, we analyzed five Klebsiella pneumoniae isolates which were consecutively isolated from a patient and showed a gradual increase in the AMR level. By genome sequencing and other analyses, we show that the first isolate was the antecedent of the later isolates and that they gained increased levels of antimicrobial resistance leading to multidrug resistance (MDR) by stepwise changes in the expression of outer membrane proteins. The isolates showing higher levels of MDR lost major porins but still colonized the patient’s gut, suggesting that the deleterious effects of porin loss were compensated for by the mutations in hexuronate metabolism-related genes and *atpG*, which were commonly detected in the MDR isolates.

## INTRODUCTION

The emergence and dissemination of antimicrobial-resistant (AMR) microorganisms are global problems in infection control (1). Among AMR pathogens, carbapenem-resistant *Enterobacteriaceae* (CRE) are considered the bacteria for which new antibiotics are most urgently needed (2). CRE are divided into carbapenemase-producing and non-carbapenemase-producing bacteria. Most of the former acquire carbapenemase-encoding genes by horizontal gene transfer (HGT), while antimicrobial-resistance phenotypes of the latter mostly result from decreased drug permeation through the outer membrane (OM) caused by intrinsic genetic alterations (3, 4).

The OM of Gram-negative bacteria includes an asymmetric lipid bilayer and numerous outer membrane proteins (OMPs), some of which are involved in the permeation of antibiotics (5, 6). Substrate-nonspecific porins and transporters play particularly important roles in antimicrobial influx and efflux, respectively. Thus, alterations in OMPs can confer multidrug resistance (MDR) on Gram-negative bacteria. The OM is also the initial binding site of antibiotics belonging to the polymyxin family, such as colistin, which is now one of the last-line drugs available to treat multidrug-resistant pathogens. Recently, colistin-resistant bacteria which overproduce enzymes that modify lipid A and prevent colistin binding have emerged (7, 8). Thus, changes in the OM and OMPs are widespread strategies of Gram-negative bacteria to adapt to antibiotic-induced stress. However, defects in porins can induce deleterious effects on bacterial proliferation and attenuate their pathogenicity (9, 10). Therefore, to survive in hosts, multidrug-resistant bacteria may have to compensate for negative effects caused by changes in the OM and OMPs by other mutations.

We previously demonstrated that just a few genomic changes could render a Klebsiella pneumoniae (family *Enterobacteriaceae*) clone multidrug resistant by lowering the permeability of antibiotics (11). However, we were unable to clarify the evolutionary processes because we could analyze only two isolates, obtained before and after the strain became multidrug resistant, respectively. We recently obtained five extended-spectrum-β-lactamase (ESBL)-producing K. pneumoniae isolates consecutively isolated from another patient continuously exposed to antibiotics. The later isolates showed increased levels of antimicrobial resistance, and the two final isolates (obtained from the same specimen) had high resistance to multiple antibiotics. In this study, we determined the complete genome sequences of the five clonal isolates. We identified gradual accumulation of mutations, inducing changes in the OMP profile, which correlated with stepwise changes in antimicrobial susceptibility. Our data also suggest specific within-host survival strategies in the isolates that acquired multidrug-resistant phenotypes via defects in major porins.

## RESULTS

### Differences in antimicrobial susceptibility of the K. pneumoniae isolates.

**FIG 1:**
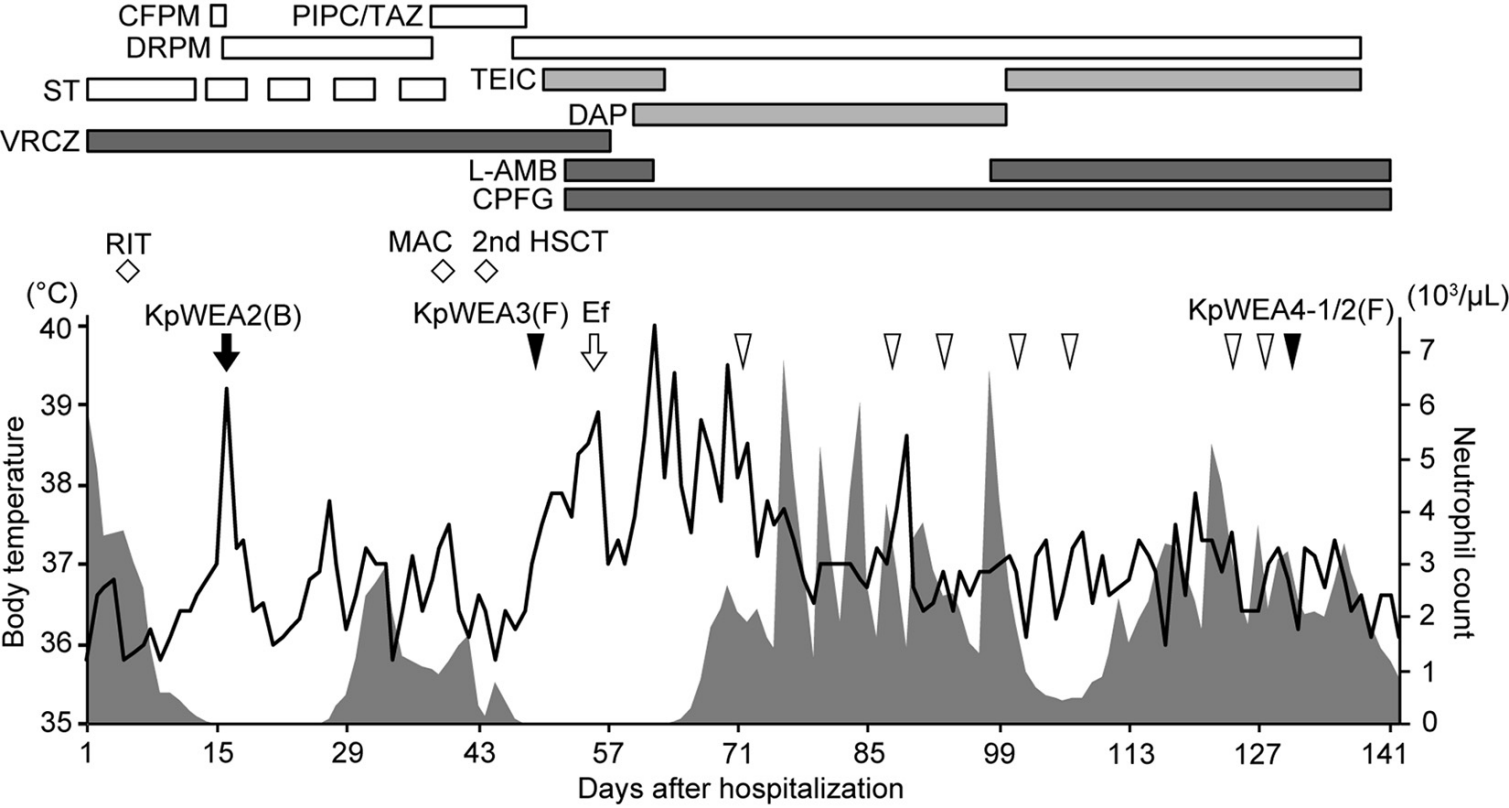
Clinical course of the patient. The arrows indicate the collection of positive blood cultures. Isolation of *bla*_CTX-M-14_-positive Klebsiella pneumoniae from fecal samples is indicated by arrowheads. KpWEA2, KpWEA3, KpWEA4-1, and KpWEA4-2 analyzed in this study were isolated at the time points indicated by the solid arrow and arrowheads. The black line and gray area indicate body temperature and absolute neutrophil counts, respectively. Antibiotics administered to the patient are indicated above the chart. RIT, remission induction therapy; MAC, myeloablative conditioning; HSCT, hematopoietic stem cell transplantation; Ef, Enterococcus faecium; (B), blood sample; (F), fecal sample. The abbreviations for and doses of antibiotics are as follows: cefepime (CFPM), 2 g every 12 h; piperacillin-tazobactam (PIPC/TAZ), 4.5 g every 8 h; doripenem (DRPM), 1 g every 8 h; sulfamethoxazole-trimethoprim (ST), 400 mg/80 mg every 48 h; teicoplanin (TEIC), 200 mg every 24 h; daptomycin (DAP), 350 mg every 24 h; voriconazole (VRCZ), 150 mg every 12 h; caspofungin (CPFG), 50 mg every 12 h; amphotericin B (l-AMB), 58 mg every 24 h.

Five K. pneumoniae isolates were consecutively isolated from a single patient, and all belonged to ST628 (Fig. 1). The first two isolates were KpWEA1 and KpWEA2; KpWEA2 was isolated 122 days after KpWEA1. These isolates exhibited the same antimicrobial susceptibility profile and were resistant to cefazolin, cefotaxime, and sulfamethoxazole-trimethoprim (Table 1). However, KpWEA3, isolated 32 days after KpWEA2, exhibited reduced susceptibility to multiple antibiotics (β-lactams other than piperacillin-tazobactam, flomoxef and carbapenems, minocycline and levofloxacin) and thus had an MDR phenotype. KpWEA4-1 and KpWEA4-2, which were isolated from a single fecal sample 84 days after the isolation of KpWEA3, showed lower susceptibility to β-lactams including piperacillin-tazobactam, flomoxef, and carbapenems. Thus, KpWEA4-1 and KpWEA4-2 were not susceptible to nearly all the β-lactams examined, minocycline, levofloxacin, or sulfamethoxazole-trimethoprim, but both were susceptible to amikacin. KpWEA4-2 additionally showed a clear decrease in susceptibility to colistin; the MIC was >16 μg/ml, while that of the other isolates was ≤2 μg/ml (note that the Clinical and Laboratory Standards Institute [CLSI] has not defined a breakpoint for colistin).

**TABLE 1:** Antimicrobial susceptibility profiles of the five K. pneumoniae isolates examined in this study^*a*^

Antimicrobial	MIC (μg/ml)				
	KpWEA1	KpWEA2	KpWEA3	KpWEA4-1	KpWEA4-2
ABPC/SBT	8	8	≥32^R^	≥32^R^	≥32^R^
PIPC/TAZ	≤0.5	≤0.5	≤0.5	≥128^R^	≥128^R^
CEZ	≥32^R^	≥32^R^	≥32^R^	≥32^R^	≥32^R^
CTX	8^R^	8^R^	≥64^R^	≥64^R^	≥64^R^
CFPM	1	1	≥32^R^	≥32^R^	≥32^R^
FMOX*	≤0.5	≤0.5	1	≥32	≥32
AZT	≤0.5	≤0.5	≥32^R^	≥32^R^	≥32^R^
IPM/CS	≤0.25	≤0.25	≤0.25	1	4^R^
MEPM	0.02	0.02	0.03	2^I^	8^R^
DRPM	0.03	0.03	0.03	2^I^	4^R^
AMK	≤1	≤1	≤1	≤1	≤1
MINO	4	4	≥16^R^	≥16^R^	≥16^R^
LVFX	0.5	0.5	≥8^R^	4^R^	4^R^
ST	≥80^R^	≥80^R^	≥80^R^	≥80^R^	≥80^R^
CL*	≤2	≤2	≤2	≤2	≥16

a The antibiotics are abbreviated as follows: ABPC/SBT, ampicillin-sulbactam; PIPC/TAZ, piperacillin-tazobactam; CEZ, cefazolin; CTX, cefotaxime; CFPM, cefepime; FMOX, flomoxef; AZT, aztreonam; IPM/CS, imipenem-cilastatin; MEPM, meropenem; DRPM, doripenem; AMK, amikacin; MINO, minocycline; LVFX, levofloxacin; ST, sulfamethoxazole-trimethoprim; CL, colistin. Superscripts of MIC values mean the following: I, intermediate; R, resistant. Breakpoints of the antimicrobials indicated by asterisks are not defined by the CLSI.

### Complete genome sequence determination of the five isolates and their genetic relationship.

**FIG 2:**
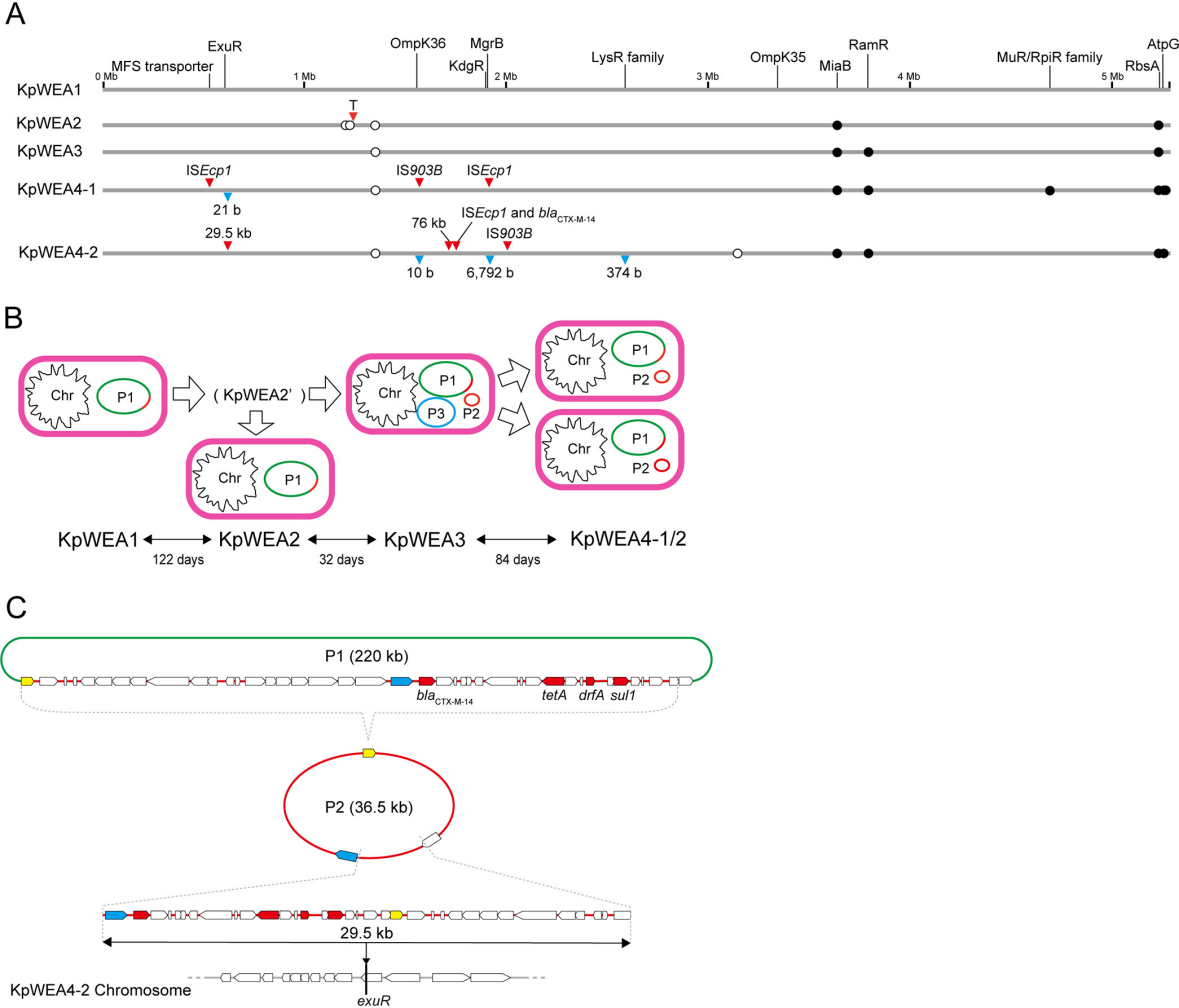
(A) Genetic alterations occurred in the chromosomes of four isolates (KpWEA2, KpWEA3, KpWEA4-1, and KpWEA4-2) derived from KpWEA1. Single-nucleotide polymorphisms (SNPs), insertions, and deletions were identified by aligning each chromosome to that of KpWEA1. Circles indicate the locations of SNPs (closed, nonsynonymous; open, synonymous). Insertions and deletions are indicated by red and blue triangles, respectively. Coding sequences affected by these genetic alterations and OmpK35 are indicated on the KpWEA1 chromosome. See Text S1 in the supplemental material for details of the genetic alterations shown in this figure. (B) Changes in the content of plasmids/extrachromosomal elements. The interval between the isolation of each isolate is also shown. (C) The genetic structure of the P2 element and the insertion of a 29.5-kb segment from P2 into the *exuR* gene on the KpWEA4-2 chromosome. The relationship between P1 and P2 is also shown. Annotation was performed using dfast v.1.2.6 with default parameters. The IS*26* transposase gene is indicated in yellow, and IS*Ecp1* in blue. Antimicrobial-resistance genes identified by ResFinder 3.2 are indicated in red.

To elucidate the genetic relationships between the five isolates, we determined their complete genome sequences. The chromosome lengths of KpWEA2 and KpWEA3 were identical to or only 1 bp different from that of KpWEA1, while those of KpWEA4-1 and KpWEA4-2 were 4,367 bp and 102,416 bp longer than that of KpWEA1, respectively (Table 2 and Fig. 2A). There were five single-nucleotide polymorphisms (SNPs) between the KpWEA1 and KpWEA2 chromosomes and four SNPs between the KpWEA1 and KpWEA3 chromosomes (Fig. 2A and see Table S2 in the supplemental material). Two of the five SNPs detected in KpWEA2 were unique to this isolate among the five genomes sequenced in this study, and three were shared by KpWEA3, KpWEA4-1, and KpWEA4-2. One of the four SNPs identified in KpWEA3 was shared by KpWEA4-1 and KpWEA4-2. Notably, KpWEA4-1 and KpWEA4-2 contained three and two additional SNPs, respectively, compared with KpWEA3, but the SNP sets newly acquired by KpWEA4-1 and KpWEA4-2 were different. These results indicate that (i) KpWEA2, KpWEA3, KpWEA4-1, and KpWEA4-2 were descendants of KpWEA1; (ii) KpWEA2 and KpWEA3 emerged from a common hypothetical intermediate; and (iii) KpWEA4-1 and KpWEA4-2 independently emerged from KpWEA3 (Fig. 2B).

**TABLE 2:** The complete genome sequences obtained in this study

Isolate	Chr^*a*^	P1	P2	P3	Accession no.
KpWEA1	5,287,432	220,075			AP024568-9
KpWEA2	5,287,433	220,075			AP024570-1
KpWEA3	5,287,432	220,075	36,504	110,870	AP024572-5
KpWEA4-1	5,291,799	220,075	36,504		AP024576-8
KpWEA4-2	5,389,848	220,075	36,504		AP024579-81

a Chr, chromosome.

10.1128/mSphere.00734-21.6 TABLE S2 SNPs in the five K. pneumoniae isolates. Download Table S2, DOCX file, 0.01 MB. Copyright © 2021 Yoshino et al. 2021 Yoshino et al. https://creativecommons.org/licenses/by/4.0/ This content is distributed under the terms of the Creative Commons Attribution 4.0 International license.

10.1128/mSphere.00734-21.1 TEXT S1 Method of intracellular ATP measurement. Download Text S1, DOCX file, 0.01 MB. Copyright © 2021 Yoshino et al. 2021 Yoshino et al. https://creativecommons.org/licenses/by/4.0/ This content is distributed under the terms of the Creative Commons Attribution 4.0 International license.

### Plasmids and their contributions to antimicrobial-resistance.

We identified three plasmids/extrachromosomal elements named P1, P2, and P3 in the five isolates (Table 2 and Fig. 2B). P1 was a 220-kb plasmid containing the IncFIB(K) and IncFII(pKP91) replicons and was present in all the isolates. P2 (36.5 kb) was acquired by KpWEA3 and inherited by KpWEA4-1 and KpWEA4-2. P3 (110 kb) was present only in KpWEA3.

The presence of *bla*_CTX-M-14_ encoding ESBL, *sul1*, and *dfrA* on the P1 genome explained the resistance of KpWEA1 and KpWEA2 to cefazolin, cefotaxime, and sulfamethoxazole-trimethoprim (Tables 1 and 3). Plasmid P1 additionally carried *tet*(A) and *qnrS1*, which might be responsible for the slightly high MICs of minocycline and levofloxacin toward KpWEA1 and KpWEA2 (Tables 1 and 3). The genome sequence of P1 was highly conserved in the five isolates with only one synonymous SNP in an IS*26*-carried gene detected in KpWEA4-2.

**TABLE 3:** Antimicrobial resistance genes found in each isolate

Antimicrobial	Resistance gene	Locus on each genome		
		KpWEA1/2	KpWEA3/4-1	KpWEA4-2
Aminoglycoside	*aac(3)*-*Iid*	P1	P1	P1
β-Lactam	*bla*_CTX-M-14_	P1	P1, P2	Chr, P1, P2
	*bla*_LAP-2_	P1	P1	P1
	*bla*_SHV-26/78/98/145/179/194/199_	Chr	Chr	Chr
Fosfomycin	*fosA*	Chr	Chr	Chr
Tetracycline	*tet*(A)	P1	P1, P2	Chr, P1, P2
Quinolone	*oqxA*	Chr	Chr	Chr
	*oqxB*	Chr	Chr	Chr
	*qnrS1*	P1	P1	P1
Sulfonamide	*sul1*	P1	P1, P2	Chr, P1, P2
Trimethoprim	*dfrA1*	P1	P1, P2	Chr, P1, P2

P2 contained no known plasmid replicons, and its sequence was identical to a part of P1 where the above-mentioned antimicrobial resistance genes except *qnrS1* are located (Table 3 and Fig. 2C). Moreover, a 29.5-kb segment of P2 was integrated into the chromosome of KpWEA4-2 (Fig. 2C). Despite its unusual features, we confirmed that P2 exists as a circular DNA by an experiment employing exonuclease V treatment of extrachromosomal DNA of KpWEA4-2 (Fig. S1). Thus, P2 appears to be a plasmid-like extrachromosomal element derived from P1 by some unknown mechanism. Further investigations are required to understand the nature of this element and the mechanisms of its generation and maintenance.

10.1128/mSphere.00734-21.2 FIG S1 Analysis of circular extrachromosomal DNAs in KpWEA4-2. DNA was extracted from KpWEA4-2 by alkaline lysis with SDS and analyzed by 0.3% agarose gel electrophoresis. The samples in each lane were treated as follows: left, no treatment; middle, digested by XbaI and SpeI; and right, digested by XbaI/SpeI and treated with exonuclease V. Any circular DNA molecules remaining in the sample after XbaI/SpeI digestion would be resistant to exonuclease V treatment. Note that while the sequence of plasmid P1 contained cleavage sites for XbaI and SpeI, that of P2 contained no such cleavage sites. Thus, the presence of DNA bands in the right lane indicates that circular DNA molecules of P2 are present in KpWEA4-2. The structural difference between the three bands observed (including a faint band) is unknown, but they may represent covalently closed or open circular molecules, and/or dimers. Download FIG S1, TIF file, 1 MB. Copyright © 2021 Yoshino et al. 2021 Yoshino et al. https://creativecommons.org/licenses/by/4.0/ This content is distributed under the terms of the Creative Commons Attribution 4.0 International license.

P3, found in KpWEA3, contained an IncFIB(pKPHS1) replicon and 113 protein-coding sequences (CDSs). Of these, none was related to antimicrobial resistance, and 99 were phage related as predicted by PHASTER. Thus, P3 is a so-called plasmid prophage.

These findings indicate that although the presence of plasmid P1 explained the AMR phenotype of KpWEA1 and KpWEA2, the higher level of resistance to multiple antibiotics acquired by their descendants, such as the resistance or increased resistance to ampicillin-sulbactam, cefotaxime, cefepime, aztreonam, minocycline, and levofloxacin acquired by KpWEA3; the resistance or intermediate resistance to piperacillin-tazobactam and carbapenems of KpWEA4-1/2; and the colistin resistance of KpWEA4-2 (Table 1), should be attributed to alterations of chromosomal sequences. Note that although the duplication and translocation of a 76-kb segment occurred specifically in the KpWEA4-2 chromosome compared with the other four isolates, the segment contained no genes apparently related to antimicrobial resistance (Fig. S2).

10.1128/mSphere.00734-21.3 FIG S2 Duplication and translocation of a 76-kb chromosomal fragment in the KpWEA4-2 chromosome. Annotation was performed using dfast v.1.2.6 with default parameters. Putative operons are indicated by different colors. Note that although the *ompK36* gene (indicated in red) is located in this fragment, the gene has been inactivated because of a 10-bp deletion in the CDS. Download FIG S2, TIF file, 1 MB. Copyright © 2021 Yoshino et al. 2021 Yoshino et al. https://creativecommons.org/licenses/by/4.0/ This content is distributed under the terms of the Creative Commons Attribution 4.0 International license.

### Stepwise change of OMP expression and correlated elevation of antimicrobial resistance.

**FIG 3:**
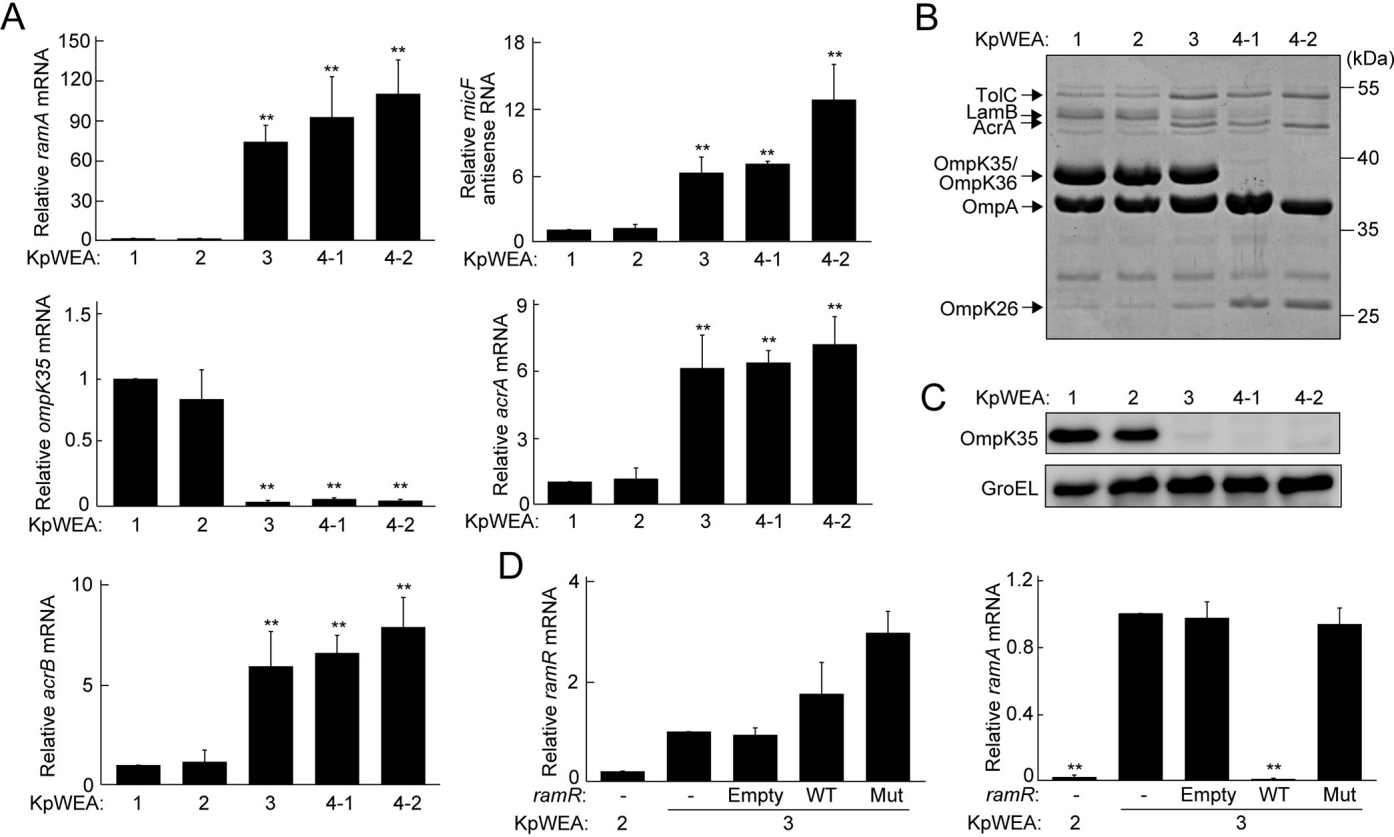
(A) Relative expression levels of *ramA* mRNA, *micF* antisense RNA, *ompK35* mRNA, and *acrA/B* mRNA in each isolate compared with those in KpWEA1. The values represent the mean ± standard deviation (SD) from three independent experiments. **, *P* < 0.01 compared with KpWEA1. (B) SDS-PAGE analysis of outer membrane proteins (OMPs) from each isolate. The proteins identified by nano-flow liquid chromatography coupled to tandem mass spectrometry are indicated. (C) Detection of OmpK35 in whole-cell extracts by immunoblotting. GroEL was used as an internal control. (D) Relative mRNA levels of *ramR* and *ramA* in KpWEA2, KpWEA3 containing no plasmid expression vector (−), KpWEA3 containing the empty plasmid expression vector (Empty), KpWEA3 containing a plasmid expressing K. pneumoniae ATCC 13883-derived *ramR* (WT), and KpWEA3 containing a plasmid expressing the KpWEA3 *ramR* allele (Mut), compared with those in KpWEA3(−). The values represent the mean ± SD from three independent experiments. **, *P* < 0.01 compared with KpWEA3(−).

In *Enterobacterales*, reduced membrane permeability can be involved in lowered susceptibilities to a broad range of antibiotics (12). Among the four SNPs found in KpWEA3, one nonsynonymous SNP in *ramR* (c.124G>A, Gly42Arg) was newly acquired by this isolate compared with KpWEA2 (Fig. 2A and Table S2). RamR is a TetR family transcriptional repressor of *ramA*; *ramA* encodes a global transcriptional activator (13). Among the genes governed by RamA, *micF* is an antisense RNA complementary to *ompK35*; *ompK35* encodes one of the two major porins of K. pneumoniae; *micF* attenuates the mRNA stability of *ompK35* and its translation (14). RamA also directly regulates the expression of components of the multidrug transporter AcrAB-TolC (13). In KpWEA3, we found that the expression of *ramA* was significantly enhanced compared with that in KpWEA1/2 (Fig. 3A), even though KpWEA3 showed a higher expression level of *ramR* (Fig. 3D). Consistent with this, the expression of *micF* and *ompK35* was significantly up- and downregulated, respectively, and that of *acrA/B* was upregulated, in KpWEA3 compared with KpWEA2 (Fig. 3A). Loss of the OmpK35 protein and increased amounts of AcrA and TolC were also confirmed in KpWEA3 (Fig. 3B and C). To examine whether the SNP in *ramR* is responsible for the overexpression of *ramA*, we analyzed the *ramA* mRNA levels in KpWEA3 derivatives in which the wild-type or the *ramR* allele of KpWEA3 was overexpressed from a plasmid (Fig. 3D). *ramA* expression was completely suppressed by overexpression of wild-type *ramR* but not by overexpression of the variant found in KpWEA3, indicating that the RamR protein of KpWEA3 lost its function as a repressor of *ramA* because of the Gly42Arg substitution.

OmpK36 is another major porin of K. pneumoniae and is involved in the influx of several antibiotics (5). As shown in Fig. 2A, an insertion of IS*903B* and a 10-bp deletion were found in the *ompK36* gene of KpWEA4-1 and KpWEA4-2, respectively, but not in KpWEA3. Consistent with this, OmpK36 was not detected in KpWEA4-1 or KpWEA4-2 (Fig. 3B).

Altogether, our analyses revealed that a stepwise change in OMP expression occurred in the descendants of KpWEA1. The loss of OmpK35 and increased production of AcrAB-TolC first occurred in KpWEA3 by a loss-of-function mutation of *ramR*, and then the loss of OmpK36 occurred additionally in KpWEA4-1 and KpWEA4-2, but by different mechanisms in the two isolates. These changes correlated with the resistance/lowered susceptibility of KpWEA3 to a broad range of antimicrobials compared with KpWEA1/2 and the further elevation of antimicrobial resistance in KpWEA4-1 and KpWEA4-2.

### Colistin resistance caused by *mgrB* deletion.

**FIG 4:**
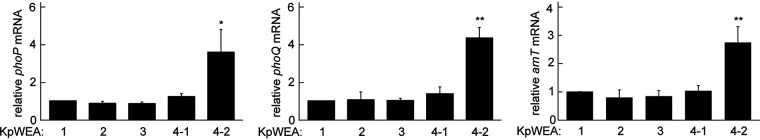
(A) Relative mRNA expression levels of *phoP* (left), *phoQ* (middle), and *arnT* (right) in the five isolates compared with those in KpWEA1. The values represent the mean ± SD from three independent experiments. *, *P* < 0.05, and **, *P* < 0.01, compared with KpWEA1.

Although the patient was not administered colistin, the MIC of colistin toward KpWEA4-2 was high (≥16 μg/ml; Table 1). Colistin resistance is mediated by modification of the phosphate moiety of lipid A with 4-amino-4-deoxy-l-arabinoseamine (l-Ara4N) and/or phosphoethanolamine, which can be introduced by chromosomal mutations and/or HGT (8). MgrB is a negative regulator of the PhoP/Q two-component regulatory system, and the loss of MgrB is frequently associated with colistin resistance in K. pneumoniae clinical isolates (15). In the absence of MgrB, the PhoP/Q system is activated and upregulates the *arn* operon that encodes enzymes for the l-Ara4N modification. As shown in Fig. 2A, the *mgrB* gene was lost by a 6,792-bp deletion in KpWEA4-2 compared with KpWEA1. We confirmed the increased expression of *phoP/Q* and also *arnT* in the *arn* operon in KpWEA4-2 (Fig. 4). Therefore, the deletion of *mgrB* presumably increased the amount of l-Ara4N-modified lipid A, which is responsible for the high MIC of colistin toward KpWEA4-2.

### Other genetic changes in KpWEA4-1 and KpWEA4-2: different mutations affecting similar functions.

KpWEA4-1 and KpWEA4-2 independently emerged from KpWEA3, and in addition to the above-mentioned inactivation of *ompK36*, they acquired different sets of additional mutations inducing amino acid substitutions and gene deletions/disruptions (Table 4). KpWEA4-1 acquired three nonsynonymous SNPs, three insertions, and one small deletion, which affected six CDSs in total, compared with KpWEA3. The mutations acquired by KpWEA4-2 changed 17 CDSs via one nonsynonymous SNP, three insertions, and three deletions, compared with KpWEA3. Note that the numbers of synonymous SNPs and intergenic insertions/deletions newly found in KpWEA4-1 or KpWEA4-2 were 0 and 2, respectively, compared with KpWEA3. Compared with KpWEA4-1, more drastic changes of the chromosome occurred in KpWEA4-2, and while both isolates exhibited lower growth rates than KpWEA1/2/3, the growth rate of KpWEA4-2 was lower than that of KpWEA4-1 (Fig. S3).

**TABLE 4:** Summary of genetic changes inducing amino acid substitutions and gene deletions/disruptions that were newly found in KpWEA4-1 and KpWEA4-2 compared with KpWEA3^*a*^

CDS (locus tag)	SNP, insertion, or deletion	
	KpWEA4-1	KpWEA4-2
MFS transporter (MAKP3_04830)	Ins (IS*Ecp1*)	
GntR family transcriptional regulator ExuR (MAKP3_05840)	Del (21 bp)	Ins (P2-derived element)
Outer membrane protein OmpK36 (MAKP3_14600)	Ins (IS*903B*)	Del (10 bp)
Polysaccharide export protein Wza (MAKP3_15860)		Ins (chromosome-derived element)
Glycosyl transferase RfaB (MAKP3_16070)		Ins (IS*Ecp1* and *bla*_CTX-M-14_)
IclR family transcriptional regulator KdgR (MAKP3_17660)	Ins (IS*Ecp1*)	Del (6,792 bp)
Hypothetical protein (MAKP3_17670)		Del (6,792 bp)
MgrB (MAKP3_17680)		Del (6,792 bp)
Hypothetical protein (MAKP3_17690)		Del (6,792 bp)
Cold shock-like protein CspC (MAKP3_17700)		Del (6,792 bp)
Cell division protein FtsI (MAKP3_17710)		Del (6,792 bp)
23S rRNA [guanine(745)-N(1)]-methyltransferase (MAKP3_17720)		Del (6,792 bp)
Putative manganese efflux pump MntP (MAKP3_17730)		Del (6,792 bp)
UPF0266 membrane protein (MAKP3_17740)		Del (6,792 bp)
PTS mannose transporter subunit IID (MAKP3_17750)		Del (6,792 bp)
Hypothetical protein (MAKP3_24060)		Del (374 bp)
LysR family transcriptional regulator (MAKP3_24070)		Del (374 bp)
DNA-binding transcriptional regulator RpiR (MAKP3_43790)	SNP (Met185Lys)	
AtpG F_o_F_1_-type ATP synthase, gamma subunit AtpG (MAKP3_48890)	SNP (Ser145Tyr, Leu146Arg)	SNP (Ile150Asn)

a Abbreviations: MFS, major facilitator superfamily; Del, deletion; Ins, insertion; PTS, phosphotransferase system.

10.1128/mSphere.00734-21.4 FIG S3 Growth curves of each isolate. Overnight culture was diluted with fresh lysogeny broth and adjusted to an optical density (OD) of 0.01 at 600 nm and then cultured at 37°C. The OD_600_ of cultures was measured every 10 min for 6 h using an Epoch 2 spectrophotometer (BioTek). Download FIG S3, TIF file, 0.2 MB. Copyright © 2021 Yoshino et al. 2021 Yoshino et al. https://creativecommons.org/licenses/by/4.0/ This content is distributed under the terms of the Creative Commons Attribution 4.0 International license.

**FIG 5:**
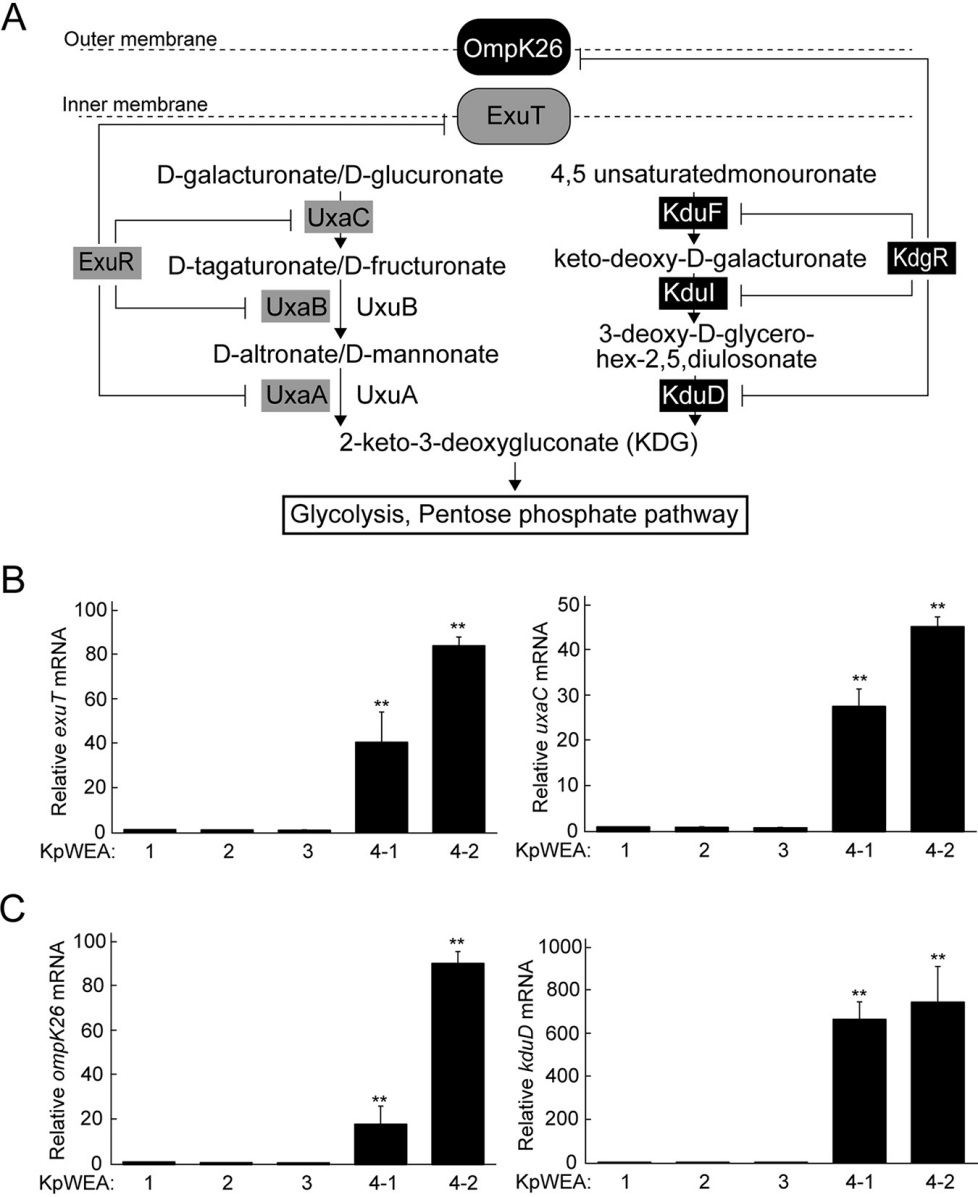
(A) The bacterial isomerase pathway of hexuronate use and the regulation of the production of each protein involved in the pathway. ExuR represses the expression of *exuT*, *uxaC*, *uxaB*, and *uxaA*. KdgR represses the expression of *ompK26*, *kduF*, *kduI*, and *kduD*. (B and C) Relative mRNA expression levels of *exuT*, *uxaC*, *ompK26*, and *kduD* in each isolate compared with those in KpWEA1. The values represent the mean ± SD from three independent experiments. **, *P* < 0.01, compared with KpWEA1.

As shown in Table 4, the two isolates (KpWEA4-1 and KpWEA4-2) acquired different mutations in each of the *exuR*, *kdgR*, and *atpG* genes. *exuR* and *kdgR* encode repressors that regulate the expression of homologous genes involved in the metabolism of hexuronates (Fig. 5A) (16). In KpWEA4-1, the *exuR* and *kdgR* genes were disrupted by a 21-bp deletion and an IS*Ecp1* insertion, respectively. In KpWEA4-2, they were disrupted by an IS*Ecp1* insertion and deleted as part of a 6,792-bp deletion, respectively (Fig. 2A and Table S3). We examined how these mutations affected the expression of genes under the control of *exuR* or *kdgR*. As expected, the expression of *exuT* and *uxaC*, both of which contain an ExuR binding site in the upstream noncoding region, was significantly upregulated in both isolates (Fig. 5B). The expression of *ompK26* and *kduD*, both of which contain a KdgR-binding site, was also upregulated in both isolates, and increased production of OmpK26 was observed (Fig. 3B and Fig. 5C). These results indicated that the loss of ExuR and KdgR, which occurred independently in KpWEA4-1 and KpWEA4-2, induced the overexpression of hexuronate metabolism-related genes, and thus, it is likely that both isolates have an increased capability to use hexuronates as carbon sources.

10.1128/mSphere.00734-21.7 TABLE S3 Summary of insertions, deletions, and SNPs. Download Table S3, DOCX file, 0.02 MB. Copyright © 2021 Yoshino et al. 2021 Yoshino et al. https://creativecommons.org/licenses/by/4.0/ This content is distributed under the terms of the Creative Commons Attribution 4.0 International license.

The *atpG* gene encodes an essential component of F_o_-F_1_-ATPase. KpWEA4-1 and KpWEA4-2 possessed different amino acid substitutions in AtpG, but the substitutions occurred at very close positions (Table 4). To assess ATP synthesis activity, we measured the intracellular ATP in each isolate; KpWEA4-1 showed no decrease in the ATP level, but the level in KpWEA4-2 was lower than that in the other isolates (Table S4). Thus, it is currently unknown whether or how the mutations in *atpG* affect the activity of F_o_-F_1_-ATPase.

10.1128/mSphere.00734-21.8 TABLE S4 Relative intracellular ATP levels in each isolate. Download Table S4, DOCX file, 0.01 MB. Copyright © 2021 Yoshino et al. 2021 Yoshino et al. https://creativecommons.org/licenses/by/4.0/ This content is distributed under the terms of the Creative Commons Attribution 4.0 International license.

Collectively, three genes, *exuR*, *kdgR*, and *atpG*, were differently altered in KpWEA4-1 and KpWEA4-2, but the mutations of these genes emerged independently in the two isolates, and the genes governed by ExuR and KdgR were notably upregulated in both isolates, suggesting that these alterations were positively selected to allow these isolates to survive within the patient.

## DISCUSSION

Here, we analyzed five K. pneumoniae ST628 isolates that were consecutively isolated from a single patient who was continuously exposed to multiple antibiotics. The isolates showed different antimicrobial resistance profiles. We confirmed that four of the five isolates were derived from the first isolate, KpWEA1. Although the genome of the second isolate, KpWEA2, was nearly identical to that of KpWEA1, genetic alterations lowering antimicrobial permeation accumulated in the three later isolates. The third isolate, KpWEA3, which showed lower susceptibility to multiple antimicrobials than KpWEA2, acquired a nonsynonymous substitution in *ramR* (Gly42Arg in helix α3). RamR binds to the upstream region of *ramA* via its N terminus (helix-turn-helix motif: α1-α3) and represses *ramA* expression (17). The substitution located in the α3 region could induce a conformational change in the DNA-binding domain of RamR. This mutation destroyed the repressor function of the protein, leading to the loss of OmpK35 production. A previous study revealed that a strain overproducing RamA showed increased tolerance to cephalosporins, tetracyclines, and fluoroquinolones (13). Consistent with this, the susceptibilities to some β-lactams, levofloxacin, and minocycline decreased in KpWEA3 compared with those in KpWEA2. The final isolates, KpWEA4-1 and KpWEA4-2, lost OmpK36 by different mechanisms and showed a further decrease in antimicrobial susceptibility. Several studies have shown that defects in the major porins increase the MICs of carbapenems toward ESBL-expressing *Enterobacterales* (4, 18, 19). Because the patient was given doripenem for a long time before the isolation of KpWEA4-1/2, subclones lacking OmpK36 could have been selected in the patient’s gut. A recent study reported that by overexpressing OmpK37 and OmpK38, *ompK35*/*ompK36* double-knockout K. pneumoniae became less resistant to some β-lactams (20). This result suggests a possibility that OmpK37 and OmpK38 may also be involved in drug permeation, but we did not find any genetic changes in the *ompK37* and *ompK38* genes between the isolates analyzed in this study. A marked phenotypic difference between KpWEA4-1 and KpWEA4-2 was the notably slower growth of KpWEA4-2 than of KpWEA4-1. The *ftsI* gene, which encodes an essential component of the divisome, was deleted from KpWEA4-2 (but not KpWEA4-1 or the other isolates). A decreased cellular ATP content was also observed in KpWEA4-2 compared with the other isolates. These genetic/physiological changes may be responsible for the slower growth of KpWEA4-2.

Since major porins, such as OmpK35 and OmpK36, are involved not only in the uptake of hydrophilic or protonated hydrophobic drugs but also in the diffusion of sugars (21), their loss results in the restriction of nutrient uptake and affects bacterial growth (9). OmpK26 is a homologue of KdgM, and its expression is regulated by KdgR (22). Although specific substrates of OmpK26 are yet to be identified, KdgM is known as a specific porin for oligogalacturonate, a metabolite of pectin (23). Use of pectin and its metabolites by *Enterobacterales* is largely unexplored, but recent studies revealed that some human gut-colonizing bacteria such *Bacteroides* can catabolize diet-derived plant pectin, suggesting that other bacteria in the gut can use galacturonate by microbial cross-feeding (24, 25). Thus, the potentially improved ability of KpWEA4-1 and KpWEA2 to use galacturonate as a carbon source may compensate to some extent for their defect in the major porins, and this may represent a specific strategy of KpWEA4-1/2-like mutants to survive in the human gut. The duplication of a 76-kb chromosomal segment containing multiple metabolism-related genes (as observed in KpWEA4-2) may also contribute to the survival of this isolate in the gut, but further analyses are required to better understand the survival strategies of major-porin-deficient strains in the human body.

Within-host evolution of AMR in K. pneumoniae by acquisition of an MDR plasmid and a nonsense mutation in *ompK36* gene has recently been reported by Tian et al. (26). Our present study also showed that the plasmid acquisition and the defect of OmpK36 contributed to the evolution of drug resistance, but we additionally identified the changes in hexuronate metabolism-related genes and *atpG*, which might compensate for the negative effects of the porin defect. Moreover, different genomic alterations in not only *ompK36* but also the same metabolism-related genes occurred in the two subclones (KpWEA4-1 and KpWEA4-2) derived from KpWEA3. These findings suggest the genetic changes were necessary for the subclones to survive within the patient’s gut. Interestingly, Jousset et al. analyzed 17 subclones (S1 to S17) of a KPC-producing K. pneumoniae ST258 clone, which were consecutively isolated from a single patient for over 4.5 years, and found that the last three subclones (S15, S16, and S17) each possessed an SNP in different subunits of respiratory complex I (27). The two final subclones examined in this study each had an SNP in *atpG* encoding a subunit of respiratory complex V, and both were nonsense mutations. These data strongly suggest that analyses of within-patient genomic evolution of K. pneumoniae isolates are useful to clarify not only antimicrobial resistance but also their energy metabolism shift to colonize the intestine under anaerobic conditions.

In this study, we analyzed only one or two K. pneumoniae colonies from each specimen. However, each specimen might contain more subclones having additional or different genetic changes and antimicrobial resistance that were not found in the isolates analyzed in this study. To gain further insights into the within-patient evolution of bacterial clones and their antimicrobial resistance, more systematic isolation of bacteria from each patient and analysis of more colonies from each sample will be required.

## MATERIALS AND METHODS

### Patient and isolation of K. pneumoniae.

A 61-year-old woman with relapsed acute myeloid leukemia (AML) was readmitted to Kyushu University Hospital (day 1, Fig. 1) to receive allogeneic hematopoietic stem cell transplantation (HSCT). When the patient was previously hospitalized for the first HSCT, *bla*_CTX-M-14_-positive K. pneumoniae (named isolate KpWEA1) was isolated from a fecal sample. Figure 1 shows the course of illness and treatment the patient underwent after readmission. Remission induction therapy started on day 5. Neutropenic fever developed on day 16. Soon after administration of cefepime empirically, *bla*_CTX-M-14_-positive K. pneumoniae (KpWEA2) was isolated from a blood sample (day 16 in Fig. 1 and 122 days after isolation of KpWEA1), and treatment with doripenem started. Then, the patient received myeloablative conditioning and subsequent HSCT. On day 48, administration of doripenem restarted because *bla*_CTX-M-14_-positive K. pneumoniae (KpWEA3) was isolated from a fecal sample. Although teicoplanin was additionally given from day 51 to treat persistent fever, high fever developed on day 55 and Enterococcus faecium was isolated from a blood sample. Blood cultures became negative after replacing teicoplanin with daptomycin. After that, although multiple antibiotics were continuously administered, *bla*_CTX-M-14_-positive K. pneumoniae bacteria were consistently isolated from fecal samples (open arrowheads in Fig. 1). Finally, two *bla*_CTX-M-14_-positive and carbapenem-intermediate/resistant K. pneumoniae isolates showing different colony morphologies (KpWEA4-1 and KpWEA4-2) were isolated from a fecal sample on day 132. The patient died on day 142 because of relapsed AML with central nervous system complications. The duration of the study was from July 2018 to March 2020.

### Strains, plasmids, and culture medium.

K. pneumoniae isolates were cultured in lysogeny broth (LB) at 37°C, except in antimicrobial susceptibility tests. To overexpress wild-type- or KpWEA3 (c.124G>A)-derived *ramR* in KpWEA3, expression plasmids constructed previously (11) were electroporated into the strain and transformants were selected on LB agar containing 100 μg/ml hygromycin B.

### Antimicrobial susceptibility tests.

MICs of antimicrobials were determined by the microdilution method. The results were interpreted according to the CLSI supplement M100, 27th edition (2017) (28).

### Genome analysis.

Genomic DNA was purified from overnight bacterial cultures using Genomic-tip 100/G (Qiagen). Libraries for short-read sequencing were prepared with the NEBNext Ultra II FS DNA library preparation kit (New England Biolabs [NEB]) and sequenced using Illumina MiSeq apparatus to obtain 301-bp paired-end reads. Libraries for long-read sequencing were prepared with the Rapid Barcode kit (SQK-RBK004), sequenced using an R9.4.1 flow cell on the Oxford Nanopore MinION system, and base-called using Guppy GPU v.3.4.5 (ONT Nanopore). Hybrid assembly of short and long reads was performed using Unicycler v.0.4.8 to obtain complete genome sequences (29). Annotation was performed using dfast v.1.2.6 with default parameters (30), and the annotation of *mgrB* was manually curated using the reference sequence with accession no. ACI07312.1. SNPs, insertions, and deletions were identified by aligning each chromosome to that of KpWEA1 using MUMmer4 (31). Plasmid replicons were identified using PlasmidFinder 2.1 (https://cge.cbs.dtu.dk/services/PlasmidFinder/) with default parameters. Antimicrobial-resistance genes were searched using ResFinder 3.2 (https://cge.cbs.dtu.dk/services/ResFinder/) with default parameters. Prophages were identified using PHASTER (32). The complete genome sequences are available in DDBJ/EMBL/GenBank (accession numbers in Table 2).

### Quantitative real-time PCR.

Total RNA was extracted from cells in the mid-log growth phase using NucleoSpin RNA (Clontech) according to the manufacturer’s instructions. RNA was reverse transcribed to cDNA using the PrimeScript RT reagent kit (TaKaRa Bio) and quantified by real-time PCR with TB Green Premix *Ex Taq* II (TaKaRa Bio) for *micF* and Thunderbird Probe qPCR mix (Toyobo) for other genes, using a StepOnePlus real-time PCR system (Thermo Fisher Scientific). Quantification was performed using a calibration curve and normalized to the expression level of *rrsH*. Table S1 in the supplemental material lists the primer and probe sequences.

10.1128/mSphere.00734-21.5 TABLE S1 Primers and probes. Download Table S1, DOCX file, 0.01 MB. Copyright © 2021 Yoshino et al. 2021 Yoshino et al. https://creativecommons.org/licenses/by/4.0/ This content is distributed under the terms of the Creative Commons Attribution 4.0 International license.

### OMP profiling.

Profiling of OMPs was performed as described previously (11). Briefly, after bacterial cells were disrupted by sonication, the OM was harvested by ultracentrifugation, washed with Tris buffer containing 2% *N*-lauroylsarcosine, and purified by ultracentrifugation again. OMPs were extracted from the purified OM and separated by one-dimensional sodium dodecyl sulfate-polyacrylamide gel electrophoresis (SDS-PAGE). Proteins were identified by nano-flow liquid chromatography coupled to tandem mass spectrometry. Immunoblotting was performed using anti-OmpK35 antiserum and anti-GroEL antibody (Abcam).

### Ethics.

This study was approved by the Center for Clinical and Translational Research of Kyushu University Hospital (reference 30-143).
