# Supplementary material for: Stepwise Evolution of a Klebsiella pneumoniae Clone within a Host Leading to Increased Multidrug Resistance
Source: mSphere. 2021 Nov 24;6(6):e00734-21. doi: 10.1128/mSphere.00734-21 (PMC8612250; doi:10.1128/mSphere.00734-21)
Supplement: TABLE S2 [file msphere.00734-21-st002.docx]

| 5260877 | 5260865 | 5260862 | 5244010 | 4692452 | 3793615 | 3639223 | 3145985 | 1353146 | 1217545 | 1217537 | Position in the KpWEA1 chromosome | | Table S2. SNPs in the five *K. pneumoniae* isolates examined in this study. |
| --- | --- | --- | --- | --- | --- | --- | --- | --- | --- | --- | --- | --- | --- |
| T | T | C | T | T | G | A | C | T | C | G | KpWEA1(ref) | SNP |  |
| T | T | C | C | T | G | T | C | A | T | A | KpWEA2 |  |  |
| T | T | C | C | T | A | T | C | A | C | G | KpWEA3 |  |  |
| T | G | A | C | A | A | T | C | A | C | G | KpWEA4-1 |  |  |
| A | T | C | C | T | A | T | T | A | C | G | KpWEA4-2 |  |  |
| MAKP3_48890 | MAKP3_48890 | MAKP3_48890 | MAKP3_48730 | MAKP3_43790 | MAKP3_35430 | MAKP3_34050 | MAKP3_29580 | MAKP3_12730 | MAKP3_r00060 | MAKP3_r00060 | Locus tag | |  |
| AtpG FoF1-type ATP synthase, gamma subunit AtpG | | | Ribose import ATP-binding protein RbsA | MurR/RpiR family transcriptional regulator | AcrR DNA-binding transcriptional regulator RamR | tRNA A37 methylthiotransferase MiaB | Hypothetical protein | Bifunctional malic enzyme oxidoreductase/phosphotransacetylase | 23S ribosomal RNA | | CDS | |  |
| Ile150Asn | Leu146Arg | Ser145Tyr | Lys279Glu | Met185Lys | Gly42Arg | Arg268Ser | - | - | - | - | Amino acid change | |  |
