# Supplementary material for: Stepwise Evolution of a Klebsiella pneumoniae Clone within a Host Leading to Increased Multidrug Resistance
Source: mSphere. 2021 Nov 24;6(6):e00734-21. doi: 10.1128/mSphere.00734-21 (PMC8612250; doi:10.1128/mSphere.00734-21)
Supplement: TEXT S1 [file msphere.00734-21-s0001.docx]

The five isolates were cultured with shaking at 250 rpm in nutrient broth (NB) at 37°C overnight. The cultured cells were diluted 100-fold into fresh NB and then cultured with shaking as described above until OD600 nm of the culture reached approximately 0.5. Intracellular ATP levels in each isolate were measured using BacTiter-Glo™ Reagent (Promega) at room temperature according to the manufacturer’s instructions. Numbers of cells used in the assays were determined by counting colony-forming units on lysogeny-agar plates, and relative light units for each isolate were normalized by the number of cells.
