# Supplementary figures and images for: Stepwise Evolution of a Klebsiella pneumoniae Clone within a Host Leading to Increased Multidrug Resistance
Source: mSphere. 2021 Nov 24;6(6):e00734-21. doi: 10.1128/mSphere.00734-21 (PMC8612250; doi:10.1128/mSphere.00734-21)

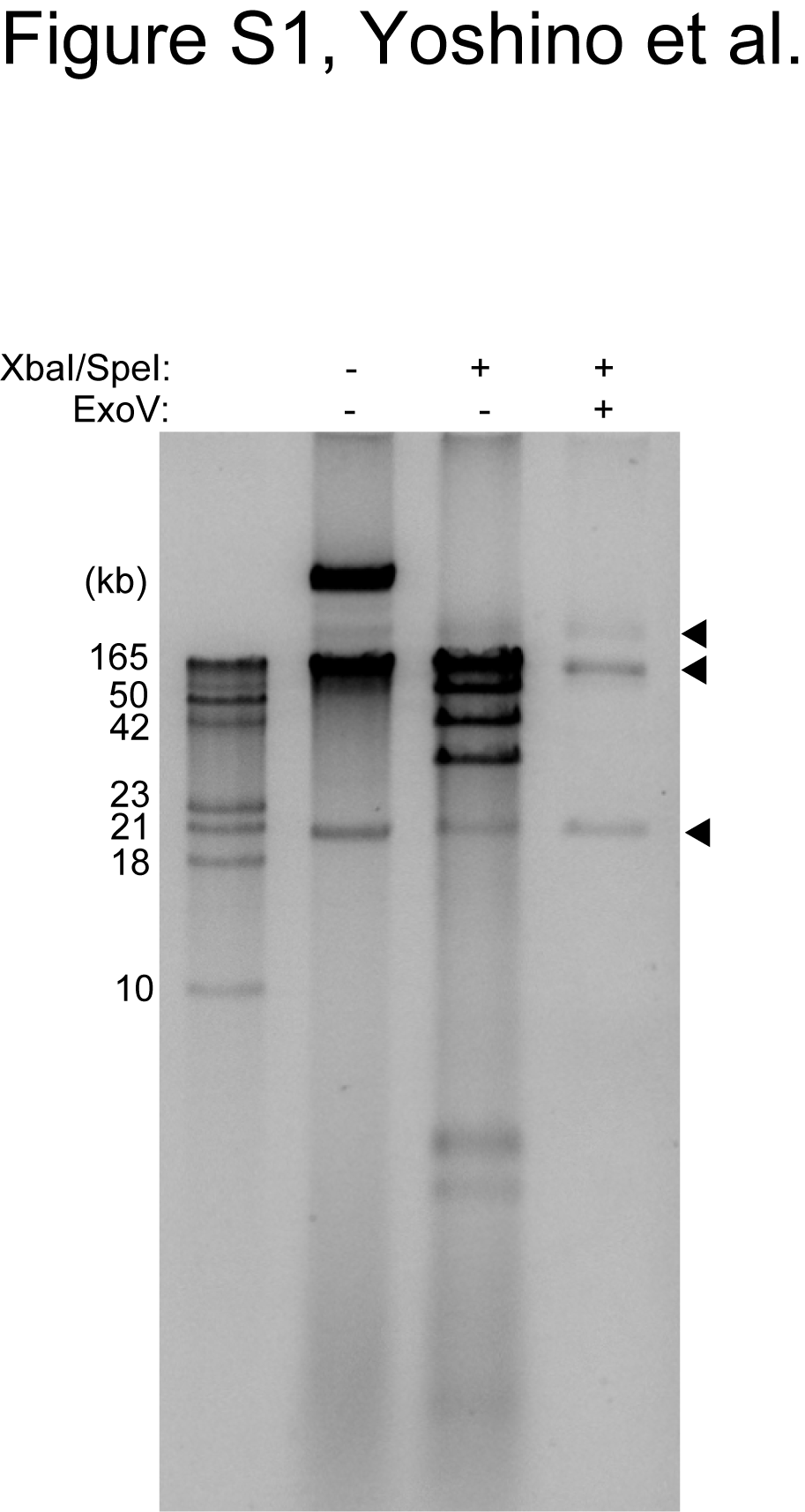

Supplement: FIG S1 [file msphere.00734-21-sf001.tif]

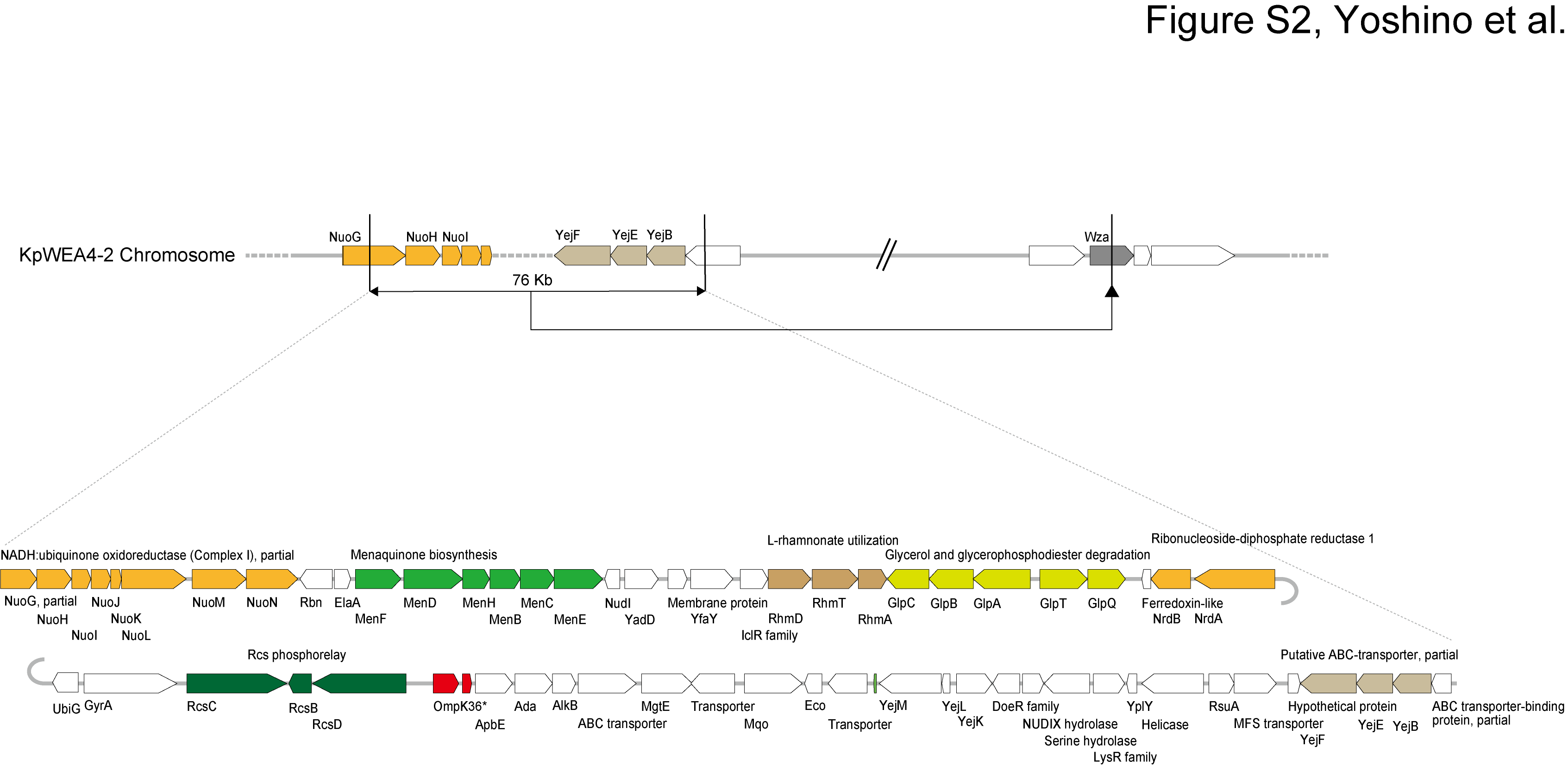

Supplement: FIG S2 [file msphere.00734-21-sf002.tif]

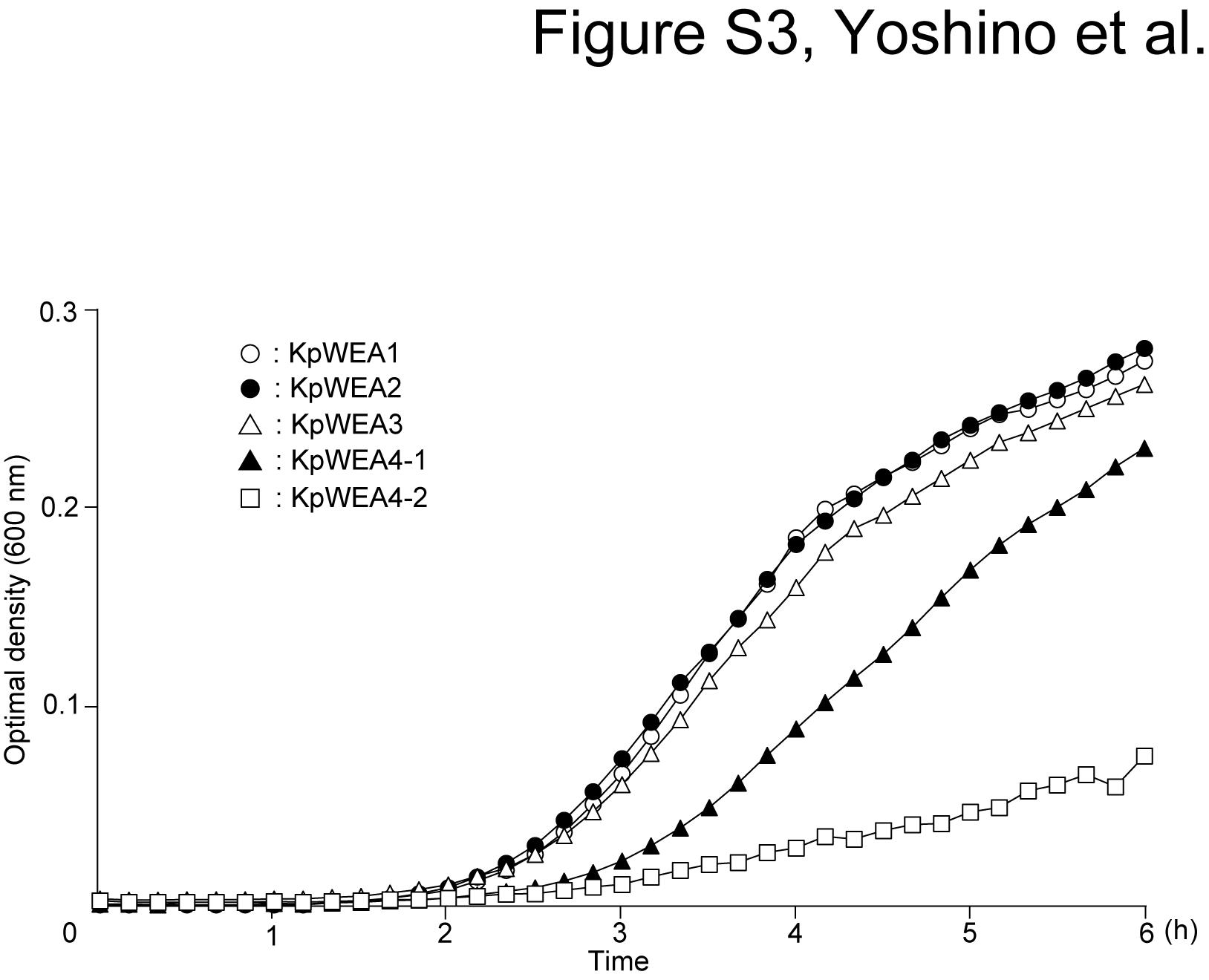

Supplement: FIG S3 [file msphere.00734-21-sf003.tif]
