# Supplementary material for: Stepwise Evolution of a Klebsiella pneumoniae Clone within a Host Leading to Increased Multidrug Resistance
Source: mSphere. 2021 Nov 24;6(6):e00734-21. doi: 10.1128/mSphere.00734-21 (PMC8612250; doi:10.1128/mSphere.00734-21)
Supplement: TABLE S3 [file msphere.00734-21-st003.docx]

| Table S3. Summary of insertions, deletions and SNPs identified in the KpWEA2, KpWEA3, KpWEA4-1, and KpWEA4-2 chromosomes using the KpWEA1 chromosome sequence as a reference. | | | | | |
| --- | --- | --- | --- | --- | --- |
| Isolate | reference (KpWEA1) | | SNP, Ins/Del | Length (b) | Detail |
|  | Position | CDS (locus_tag) |  |  |  |
| KpWEA2 | 1217537 | 23S ribosomal RNA (MAKP3_r00060) | SNP | - | G>A |
|  | 1217545 | 23S ribosomal RNA (MAKP3_r00060) | SNP | - | C>T |
|  | 1217650 | Intergene between 23S ribosomal RNA (MAKP3_r00060) and 5S ribosomal RNA (MAKP3_r00070) | Ins | 1 | T |
|  | 1353146 | Bifunctional malic enzyme oxidoreductase/phosphotransacetylase (MAKP3_12730) | SNP | - | T>A (synonymous) |
|  | 3639223 | tRNA A37 methylthiotransferase MiaB (MAKP3_34050) | SNP | - | A>T (Arg268Ser) |
|  | 5244010 | Ribose import ATP-binding protein RbsA (MAKP3_48730) | SNP | - | T>C (Lys279Glu) |
| KpWEA3 | 1353146 | Bifunctional malic enzyme oxidoreductase/phosphotransacetylase (MAKP3_12730) | SNP | - | T>A (synonymous) |
|  | 3639223 | tRNA A37 methylthiotransferase MiaB (MAKP3_34050) | SNP | - | A>T (Arg268Ser) |
|  | 3793615 | AcrR DNA-binding transcriptional regulator RamR (MAKP3_35430) | SNP | - | G>A (Gly42Arg) |
|  | 5244010 | Ribose import ATP-binding protein RbsA (MAKP3_48730) | SNP | - | T>C (Lys279Glu) |
| KpWEA4-1 | 515856 | MFS transporter (MAKP3_04830) | Ins | 1,661 | IS*Ecp1*(IS*1380* family) |
|  | 606055-606075 | GntR family transcriptional regulator ExuR (MAKP3_05840) | Del | 21 | - |
|  | 1353146 | Bifunctional malic enzyme oxidoreductase/phosphotransacetylase (MAKP3_12730) | SNP | - | T>A (synonymous) |
|  | 1555810 | Outer membrane protein OmpK36 (MAKP3_14600) | Ins | 1,066 | IS*903B* (IS*5* family) |
|  | 1901293 | IclR family transcriptional regulator KdgR (MAKP3_17660) | Ins | 1,661 | IS*Ecp1*(IS*1380* family) |
|  | 3639223 | tRNA A37 methylthiotransferase MiaB (MAKP3_34050) | SNP | - | A>T (Arg268Ser) |
|  | 3793615 | AcrR DNA-binding transcriptional regulator RamR (MAKP3_35430) | SNP | - | G>A (Gly42Arg) |
|  | 4692452 | DNA-binding transcriptional regulator RpiR (MAKP3_43790) | SNP | - | T>A (Met185Lys) |
|  | 5244010 | Ribose import ATP-binding protein RbsA (MAKP3_48730) | SNP | - | T>C (Lys279Glu) |
|  | 5260862 | AtpG FoF1-type ATP synthase, gamma subunit AtpG (MAKP3_48890) | SNP | - | C>A (Ser145Tyr) |
|  | 5260865 | AtpG FoF1-type ATP synthase, gamma subunit AtpG (MAKP3_48890) | SNP | - | T>G (Leu146Arg) |
| KpWEA4-2 | 605827 | GntR family transcriptional regulator ExuR (MAKP3_05840) | Ins | 29,520 | 5-b duplication (TAAAT) and 29,515 bp insertion derived from P2 (position: 20,481-36,504, 1-13,491) |
|  | 1353146 | Bifunctional malic enzyme oxidoreductase/phosphotransacetylase (MAKP3_12730) | SNP | - | T>A (synonymous) |
|  | 1556395-1556404 | Outer membrane protein OmpK36 (MAKP3_14600) | Del | 10 | - |
|  | 1707948 | Polysaccharide export protein Wza, (MAKP3_15860) | Ins | 76,066 | 4-b duplication (TCAC) and 76,062 bp insertion derived from Chr (*potition:1,508,568-1,584,639 in Kp3) |
|  | 1737250 | Glycosyl transferase RfaB (MAKP3_16070) | Ins | 2,940 | IS*Ecp1* and *bla*_CTX-M-14_ |
|  | 1901472-1908263 | IclR family transcriptional regulator KdgR (MAKP3_17660)  Hypothetical protein (MAKP3_17670) MgrB (MAKP3_17680)** Hypothetical protein (MAKP3_17690) Cold shock-like protein CspC (MAKP3_17700) Cell division protein FtsI (MAKP3_17710) 23S rRNA (guanine(745)-N(1))-methyltransferase (MAKP3_17720) Putative manganese efflux pump MntP (MAKP3_17730) UPF0266 membrane protein (MAKP3_17740) PTS mannose transporter subunit IID (MAKP3_17750) | Del | 6,792 | - |
|  | 1991429 | Intergene between hypothetical protein (MAKP3_18470) and hypothetical protein (MAKP3_18480) | Ins | 1,066 | IS*903B* (IS*5* family) |
|  | 2573747-2574120 | Hypothetical protein (MAKP3_24060) LysR family transcriptional regulator (MAKP3_24070) | Del | 374 | - |
|  | 3145985 | Hypothetical protein (MAKP3_29580) | SNP | - | C>T (synonymous) |
|  | 3639223 | tRNA A37 methylthiotransferase MiaB (MAKP3_34050) | SNP | - | A>T (Arg268Ser) |
|  | 3793615 | AcrR DNA-binding transcriptional regulator RamR (MAKP3_35430) | SNP | - | G>A (Gly42Arg) |
|  | 5244010 | Ribose import ATP-binding protein RbsA (MAKP3_48730) | SNP | - | T>C (Lys279Glu) |
|  | 5260877 | AtpG FoF1-type ATP synthase, gamma subunit AtpG (MAKP3_48890) | SNP | - | T>A (Ile150Asn) |
| * The fragment contained *ompk36* gene with the 10 bp-deletion. | | |  |  |  |
| ** Manually curated. | |  |  |  |  |
