# Supplementary material for: Stepwise Evolution of a Klebsiella pneumoniae Clone within a Host Leading to Increased Multidrug Resistance
Source: mSphere. 2021 Nov 24;6(6):e00734-21. doi: 10.1128/mSphere.00734-21 (PMC8612250; doi:10.1128/mSphere.00734-21)
Supplement: TABLE S4 [file msphere.00734-21-st004.docx]

| Table S4. Relative intracellular ATP levels in each isolate. | |
| --- | --- |
| Isolate | *RLU/10^6^ CFUs |
| KpWEA1 | 8,850±946 |
| KpWEA2 | 11,439±4635 |
| KpWEA3 | 10,084±3853 |
| KpWEA4-1 | 12,339±3885 |
| KpWEA4-2 | 6,616±2549 |
| *The values for relative light units (RLU)/10^6^ colony-forming units (CFUs) represent mean ± s.d. values from four independent experiments. | |
