# Supplementary material for: Stepwise Evolution of a Klebsiella pneumoniae Clone within a Host Leading to Increased Multidrug Resistance
Source: mSphere. 2021 Nov 24;6(6):e00734-21. doi: 10.1128/mSphere.00734-21 (PMC8612250; doi:10.1128/mSphere.00734-21)
Supplement: TABLE S1 [file msphere.00734-21-st001.docx]

| Table S1. Primers and probes used in this study. | | |  |
| --- | --- | --- | --- |
| Gene | Sense primer | Antisense primer | Probe |
| *rrsH* | atcggaattactgggcgtaaag | tagcctgccagtttcgaatg | ttcacatccgacttgacagaccgc |
| *ompK35* | gtctggaccaccaatggc | atctgagtttcgcctttcag | ccacctatgcccgtatcggcc |
| *acrA* | atcacgctacgcgctattt | gtcagggttaatcccttcttcc | aacccggatcacaccctgctac |
| *acrB* | caatacggaagagtttggca | cagacgaacctgggaacc | tcctggttcaccttcagcaggatg |
| *ramA* | cgctcaggtgattgacactat | ttgcagatgccatttcgaatac | tcgagtggattgatgacaacctgca |
| *ramR* | ggctcgtccaaagagtgaag | tgagcaactcatccttggtg | aaagcaagcgttactggaagctgc |
| *phoP* | cgtactcgtggttgaggataatgc | gagatgttcgcccagatagtagtcc | tcaccacctcaaagttcagctgca |
| *phoQ* | cgatgagcagggcaaactg | gtcgacatcggcttcaatctc | aaccggaatggctgaaacgcaatg |
| *arnT* | tcttcgccatcaccatgac | gaagataatcggcgacaggatag | tggcggatctttcgtgataagcgg |
| *uxaC* | ttataccactggacccatttagag | tgagccagcaaatcattacac | aggtaagctgttgtcgccatcca |
| *exuT* | acgctgatggaagagttacac | ccagaatatcaaggacataccc | agcctattccgctgcctataccgtt |
| *kduD* | agactatcgagcgagtaaccg | ctccagaagctgaggaatacc | tttcttagcctgaccgccgacc |
| *ompk26* | accattgagagcaacgacag | ggtgtattcatagcgatagcg | agtacagcttcgacaacggcttct |
| *micF* | tcatcattaactttatttattaccg | gaaataggggtaaacagac | - |
